# Similarity-Based Unsupervised Spelling Correction Using BioWordVec: Development and Usability Study of Bacterial Culture and Antimicrobial Susceptibility Reports

**DOI:** 10.2196/25530

**Published:** 2021-02-22

**Authors:** Taehyeong Kim, Sung Won Han, Minji Kang, Se Ha Lee, Jong-Ho Kim, Hyung Joon Joo, Jang Wook Sohn

**Affiliations:** 1 Division of Industrial Management Engineering Korea University Seoul Republic of Korea; 2 Information Computing Office Korea University Anam Hospital Seoul Republic of Korea; 3 Department of Cardiology Cardiovascular Center Korea University College of Medicine Seongbuk-gu Republic of Korea; 4 Division of Infectious Disease Department of Internal Medicine Korea University College of Medicine Seoul Republic of Korea

**Keywords:** spelling correction, natural language processing, bacteria, electronic health record

## Abstract

**Background:**

Existing bacterial culture test results for infectious diseases are written in unrefined text, resulting in many problems, including typographical errors and stop words. Effective spelling correction processes are needed to ensure the accuracy and reliability of data for the study of infectious diseases, including medical terminology extraction. If a dictionary is established, spelling algorithms using edit distance are efficient. However, in the absence of a dictionary, traditional spelling correction algorithms that utilize only edit distances have limitations.

**Objective:**

In this research, we proposed a similarity-based spelling correction algorithm using pretrained word embedding with the BioWordVec technique. This method uses a character-level N-grams–based distributed representation through unsupervised learning rather than the existing rule-based method. In other words, we propose a framework that detects and corrects typographical errors when a dictionary is not in place.

**Methods:**

For detected typographical errors not mapped to Systematized Nomenclature of Medicine (SNOMED) clinical terms, a correction candidate group with high similarity considering the edit distance was generated using pretrained word embedding from the clinical database. From the embedding matrix in which the vocabulary is arranged in descending order according to frequency, a grid search was used to search for candidate groups of similar words. Thereafter, the correction candidate words were ranked in consideration of the frequency of the words, and the typographical errors were finally corrected according to the ranking.

**Results:**

Bacterial identification words were extracted from 27,544 bacterial culture and antimicrobial susceptibility reports, and 16 types of spelling errors and 914 misspelled words were found. The similarity-based spelling correction algorithm using BioWordVec proposed in this research corrected 12 types of typographical errors and showed very high performance in correcting 97.48% (based on F1 score) of all spelling errors.

**Conclusions:**

This tool corrected spelling errors effectively in the absence of a dictionary based on bacterial identification words in bacterial culture and antimicrobial susceptibility reports. This method will help build a high-quality refined database of vast text data for electronic health records.

## Introduction

### Background

Among various industries, the medical industry produces many unstructured forms of examination reports. It is very important to establish a structured form of accurate medical documentation to provide accurate diagnoses and treatments to patients [1]. False medical information because of spelling errors can lead to medical and/or treatment errors, resulting in serious risks for patients. For example, errors in the spelling of organism names or drugs with similar spelling in bacterial culture tests have negative effects on not only the diagnosis and treatment of patients, but also the management of infectious diseases and nosocomial infections in hospitals.

While many patient electronic health records are documented in a structured form, the bacterial culture report is still stored as images or as an unrefined text data form in most hospitals. Mapping terms for bacterial identification are necessary to proceed with medical data studies, such as detection and diffusion path studies of infectious diseases. However, since large-scale clinical text data are mostly written by doctors or semiautomatic systems, there can be problems with data consistency, typographical errors, and stop words [2].

In clinical text data, the extraction-transformation-load (ETL) process for medical terms is typically performed through exact string matching of words that appear in the dictionary. However, words not present in the dictionary or severely misspelled words have difficulty matching to terms. Because medical terms are complex and field specific, this problem makes it difficult to apply the same general data refining methods [3]. Rule-based spelling correction algorithms cannot ensure the accuracy and reliability of the data because of incorrect data preprocessing. This method also has to check all test results and find the errors directly, resulting in a considerable cost problem.

### Related Work

#### Spelling Correction in the Medical Domain

It is very difficult to construct dictionaries for all medical terms and abbreviations. A related study of spelling correction algorithms specialized in medical record text data was conducted. Lai et al [4] proposed a noisy channel-based spelling check algorithm for medical text. Named entity recognition (NER) was used to achieve an error detection performance of up to 94.4% with a spelling correction accuracy of up to 88.2%, producing high performance spelling correction results in various clinical documents. Fivez et al [5,6] proposed a spelling check algorithm for clinical free text using fastText of the N-gram embedding technique. After generating misspelled words in MIMIC-III [7] to measure similarity with the candidate group that fits the context, the similarity was ranked using the Damerau-Levenshtein distance. This method suggested a way to solve the out-of-vocabulary (OOV) problem in clinical data.

#### Subword-Level Word Vector Representation

Traditional spelling correction algorithms using edit distance or pronunciation algorithms have limitations in correcting word-level issues that fit the context. There are subword-level embedding methods for learning concurrent word information to consider context understanding. FastText [8] expresses a word by the sum of the N-gram vector of the character level. The embedding method at the subword level solves the disadvantages that involve difficulty in application to languages with varying morphological changes or low frequency. This method was strong at solving the OOV problem, and accuracy was high for rare words in the word set. BioWordVec [9] learns clinical record data from PubMed and MIMIC-III clinical databases using fastText. Based on 28,714,373 PubMed documents and 2,083,180 MIMIC-III clinical database documents, the entire corpus was built. The Medical Subject Headings (MeSH) term graph was organized to create a heading sequence and to carry out word embedding based on a sequence combining MeSH and PubMed. BioWordVec provided a 200-dimensional pretrained word embedding matrix.

### Limitations With Existing Approaches

The method proposed by Lai et al [4] has a limitation in that spelling corrections are not made in the absence of a dictionary. The method proposed by Fivez et al [5,6] solves the OOV problem, but has a similar limitation in that spelling corrections are not made in the absence of a dictionary.

### Our Approach

This paper proposes a similarity-based spelling correction algorithm through pretrained word embedding in medical field data. Using the BioWordVec model of the character level, which has pretrained clinical record data from the MIMIC-III clinical database, the model progresses learning on spelling corrections end-to-end. The proposed model has the advantage of being able to make spelling corrections in the absence of a dictionary. In addition, it is effective against new types of typographical errors that may occur in the future, and it is highly utilized in the field because it uses unsupervised learning that does not require direct label assignment. We aimed to use this model to develop a spelling correction system suitable for various types of medical text data.

## Methods

### Data Set

#### Bacterial Culture and Antimicrobial Susceptibility Reports

In this study, the bacterial culture and antimicrobial susceptibility reports from Korea University Anam Hospital, Korea University Guro Hospital, and Korea University Ansan Hospital were used. The bacterial culture and antimicrobial susceptibility report data were collected for 17 years (from 2002 to 2018), and in each year, reports for 1 month were used for the experiment. In total, 180,000 items were retrieved, with 27,544 having meaningful test results. Using the self-developed rule-based ETL algorithm [10], unstructured bacterial culture and antimicrobial susceptibility reports were converted into structured text data. After preprocessing through lexical processing, such as sentence segmentation, tokenization, and stemming using regular expressions, there were 320 types of bacterial identification words in the report. Among the extracted bacterial identification words, 16 types of spelling errors and 914 misspelled words were found. Table 1 presents the typographical errors based on their occurrence.

**Table 1:** Misspelling frequency table.

Misspelling	Occurrence, n
staphylococcus	827
sstreptococcus	21
adecarboxylate	19
parpinfluenzae	18
papatyphi	7
pseudodiphthericum	6
urealyticm	5
chromogens	2
flavbacterium	2
ferentum	1
koneensis	1
ochrobacterium	1
orytihabitans	1
shingobacterium	1
stacherbrandfii	1
perosis	1

### Methodology

#### Misspelling Detection

Systematized Nomenclature of Medicine (SNOMED) clinical terms (CT) [11] is a set of systematically structured medical terms used in medical clinical documents and reports. It is the world’s largest multilingual clinical terminology system. In the corpus constructed by tokenizing the bacterial identification result reports, words that were not mapped to SNOMED CT were defined and detected as typographical errors [12].

#### Candidate Generation

Using the fastText [8] technique, prelearned word embedding was used to generate a group of corrected word candidates with high similarity considering the edit distance. In this study, the BioWordVec [9] model that was prelearned from the clinical database was used.

The number of words that were most similar, cosine similarity, and edit distance were set as hyperparameters for generating a correction candidate group. In addition, constraints for candidate words were used based on the dictionary constructed for the existing general terms, the length of the word, and the frequency of the word. In this study, the number of most similar words was set to 30, cosine was set to 0.80, and edit distance was set to 3 as hyperparameters.

Character-based spell checking algorithms were used to determine edit distances to generate or rank candidate groups. The Levenshtein edit distance [13] is the number of operations required to convert one word into another. It can find the minimum editing distance that considers the insertion, deletion, replacement, and transposition (replacement of two adjacent characters) for most spelling errors. The model proposed in this paper uses the Damerau-Levenshtein distance [14] as the edit distance. The formula is as follows:


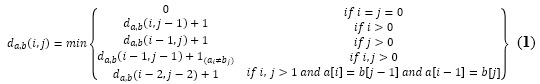


#### Candidate Ranking

The final correction word is suggested by ranking the correction candidate groups. The pretrained word embedding was learned by the fastText technique, and the vocabulary was sorted in descending order according to frequency. The methodology proposed in this study has two assumptions. First, in clinical databases, correctly spelled words may appear relatively more frequently than misspelled words [15]. Second, the larger the corpus used for learning, the greater the frequency of correctly spelled words [15]. The BioWordVec [9] model used in this research can sufficiently satisfy the above two assumptions.

The model proposed in this research limited the search for the range of the most similar words. Through a grid search, a similarity-based candidate group that considers the frequency of words was proposed [16]. After sorting the ranking of the generated correction candidate words based on similarity, typographical errors can be corrected.

### Overall Architecture

**Figure 1:**
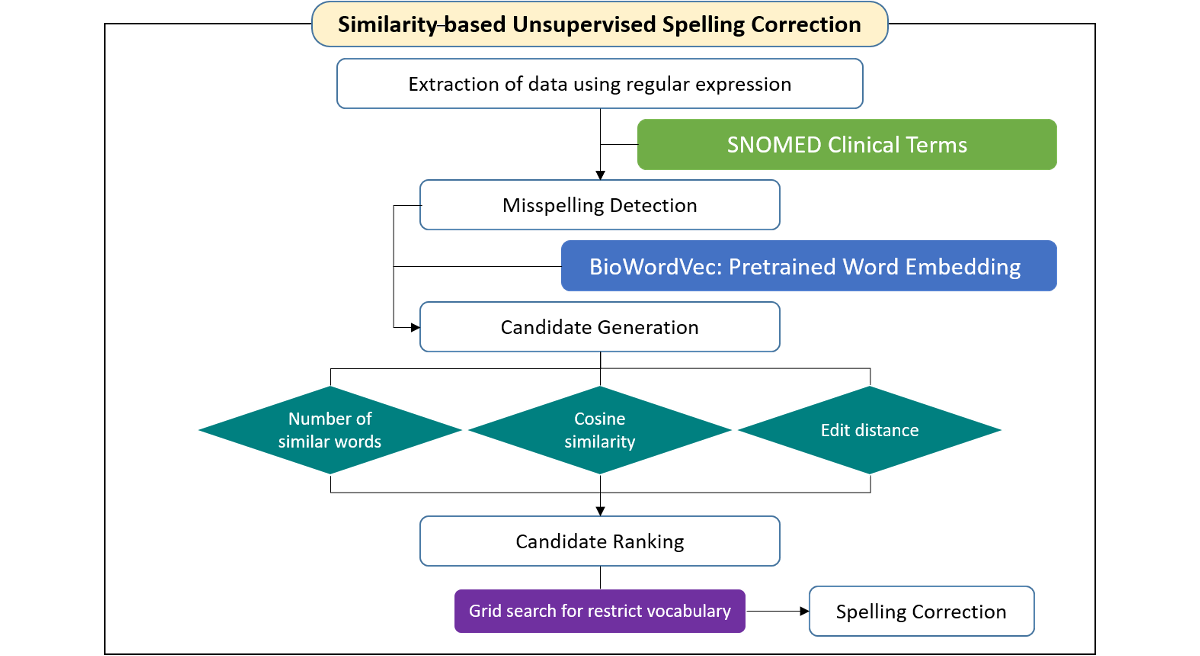
Similarity-based unsupervised spelling correction architecture. SNOMED: Systematized Nomenclature of Medicine.

Figure 1 shows the architecture of the spelling correction algorithm proposed in this paper.

## Results

### Experiments

A typographical error that appears in bacterial culture and antimicrobial susceptibility reports is a word that can be corrected within three edit distances, as shown in Table 2. Most typographical errors have a correctly spelled word within one edit distance. Therefore, in the model proposed in this study, the critical value of the editing distance for generating the correction candidate group was set to 3 or less.

**Table 2:** Correction table using edit distance.

Correction	Edit distance
stapylococcus to staphylococcus	1
sstreptococcus to streptococcus	1
adecarboxylate to adecarboxylata	1
parpinfluenzae to parainfluenzae	1
papatyphi to paratyphi	1
pseudodiphtericum to pseudodiphtheriticum	2
urealyticm to urealyticum	1
chromogens to chromogenes	1
flavbacterium to flavobacterium	1
ferentum to fermentum	1
koneensis to koreensis	1
ochrobacterium to ochrobactrum	2
orytihabitans to oryzihabitans	1
shingobacterium to sphingobacterium	1
stacherbrandfii to stackebrandtii	3
perosis to peroris	1

#### Comparison of Pretrained Embeddings

All of the pretrained word embeddings used in this study were learned based on the fastText methodology, and the corpus was constructed without distinction between spelling errors and correct spelling during learning. To compare the performance of the BioWordVec model introduced in the previous study, four pretrained embeddings provided by Facebook were used.

The following are the five pretrained embeddings: (1) BioWordVec, 200-dimensional embedding vectors learned using fastText for PubMed and MIMIC-III; (2) English word vectors, 300-dimensional embedding vectors learned using fastText for general text and from Wikipedia; (3) Crawled English subword vectors, 300-dimensional embedding vectors learned using fastText for the 2,000,000 lower words that appear in English word documents; (4) Wiki word vectors, 300-dimensional embedding vectors learned using fastText in Wikipedia; (5) Simple Wiki word vectors, 300-dimensional embedding vectors learned using fastText in Simple Wikipedia.

The cosine similarity of all models was set to 0.80 or higher, the editing distance threshold was set to 3 or less, and the most similar words were tested under the same conditions with 30 words. The evaluation index is the exact spelling of the total 16 typographical errors that appear in the bacterial assimilation report with correction rate. Table 3 shows the rate of correction for typographical errors according to pretrained embeddings.

The spelling correction algorithm using BioWordVec showed very high performance compared to the performance of the other pretrained word embedding models. The methodology proposed in this study has the advantage of being used even in the absence of a dictionary. However, it was confirmed that pretrained word embedding based on the clinical database is necessary to correct errors in the bacterial identification report.

**Table 3:** Comparison of pretrained embedding.

Pretrained embedding model	Correction rate
BioWordVec	0.75
English word vectors	0.00
Crawled English subword vectors	0.00
Wiki word vectors	0.31
Simple Wiki word vectors	0.19

### Evaluation

Through a comparative experiment as shown in Table 3, it is possible to correct typographical errors using pretrained word embedding without building a dictionary. To evaluate the performance of the model proposed in this study, its performance was compared with a rule-based spelling correction algorithm [17] using a dictionary and a situation without spelling correction. SymSpell [18] was used as a spelling correction algorithm based on the edit distance rule.

SymSpell [18] can correct typographical errors 1 million times faster than rule-based spelling correction [17] and can use existing dictionaries through a symmetric deletion spelling correction algorithm. SymSpell uses the Damerau-Levenshtein edit distance [14], which was set to 3 for the experiment under the same conditions as the model proposed in this study. SCOWL [19] and Dorland medical dictionary [20,21] were used as dictionaries for SymSpell, and a total of 100,000 correct word dictionaries were constructed.

Table 4 shows the evaluation results through the NER task that extracts the bacterial identification words. In the table, accuracy is the number of words corrected for all misspellings. Precision is the proportion of corrected words that the actual corrections match exactly. Recall is the proportion of correct corrected words among actual typographical errors. F1 score is the harmonic mean of precision and recall. SUSC (similarity-based unsupervised spelling correction) in Table 4 is the model proposed in this study.

In this study, the similarity-based spell checking algorithm SUSC using BioWordVec corrected 12 types of typographical errors and showed very high performance in correcting 97.48% (based on F1 score) of all spelling errors. Both models were able to correct frequent typographical errors, so the overall correction rate was high. However, since SymSpell only corrects certain words, the F1 score showed little difference compared with the nonspelling situation. The Dorland medical dictionary was not able to fully understand bacterial identification names for infectious diseases, and the rule-based spell checking algorithms using edit distance did not work well according to the established dictionaries. Constructing an accurate dictionary that can be used in a rule-based spell checking algorithm is very expensive and time consuming.

**Table 4:** Model performance using BioWordVec.

Model	Accuracy	Precision	Recall	F1 score
No spelling correction	0.98	0.94	0.93	0.94
SymSpell	1.00	0.94	0.94	0.94
SUSC^a^ (BioWordVec)	1.00	0.97	0.97	0.97

^a^SUSC: similarity-based unsupervised spelling correction.

#### Comparison of Similarity

Using the SUSC model proposed in this study, the degree of similarity of words depending on correction was examined. Table 5 shows the similarity of words according to whether they are corrected.

As shown in Table 5, typographical errors that were not corrected with the correct spelling have low cosine similarity with the correctly spelled word as a whole. In the case of nonword errors, which involve words that do not actually exist, most of the words were corrected accurately. Miscorrected typographical errors included real-word errors where the word actually exists but is not appropriate for grammar or context. Since real-word errors are determined to be similar in meaning to words that do not fit the situation, the cosine similarity is relatively low for the word vector to be corrected. The model proposed in this study has the advantage of quantitatively expressing the relative distance between typographical errors and correctly spelled words by utilizing the similarity between words. Through the proposed model, it is possible to compare and determine whether the error detected with the framework is actually a typographical error that can occur often.

**Table 5:** Comparison of similarity according to correction.

Change	Correction	Similarity
adecarboxylate to adecarboxylata	Corrected	0.90
flavbacterium to flavobacterium	Corrected	0.83
koneensis to koreensis	Corrected	0.87
ochrobacterium to ochrobactrum	Corrected	0.93
orytihabitans to oryzihabitans	Corrected	0.90
papatyphi to paratyphi	Corrected	0.89
parpinfluenzae to parainfluenzae	Corrected	0.86
pseudodiphtericum to pseudodiphtheriticum	Corrected	0.93
shingobacterium to sphingobacterium	Corrected	0.93
sstreptococcus to streptococcus	Corrected	0.95
stapylococcus to staphylococcus	Corrected	0.88
urealyticm to urealyticum	Corrected	0.84
chromogens to chromogenes	Not corrected	0.71
ferentum to fermentum	Not corrected	0.47
perosis to peroris	Not corrected	0.42
stacherbrandfii to stackebrandtii	Not corrected	0.59

## Discussion

It is difficult to compare our results with previous results because the system implementation and data set used in the related work are not publicly available. The model proposed in this research was capable of spelling correction through unsupervised learning, but it lacked the performance required for infrequent typographical errors and real-word errors. In addition, there was a problem of randomly setting the reference values for cosine similarity and edit distance when creating a correction candidate group. Methods should be devised to establish appropriate thresholds for hyperparameters through experiments.

This research proposes a similarity-based spelling correction algorithm using pretrained word embedding to extract correct medical terminology from unstructured text data related to infectious diseases. The suggested algorithm has the advantage of being able to check spelling and make corrections in the absence of a correct spelling dictionary. In addition, it solves the OOV problem and can modify words based on context.

As a result of the experiments conducted in this research, we were able to detect and correct spelling errors in the absence of a dictionary for bacterial terms appearing in bacterial culture and antimicrobial susceptibility reports. Our model efficiently refined and processed large medical text data. It has been proven experimentally that it is a method suitable for processing natural language involving high expertise and complexity, such as medical terminology. Ideally, the results of this research will serve as a foundation to build vast amounts of text data in electronic health records into high-quality databases.

## Abbreviations

CT: clinical terms
ETL: extract-transformation-load
MeSH: Medical Subject Headings
NER: named entity recognition
OOV: out-of-vocabulary
SNOMED: Systematized Nomenclature of Medicine
SUSC: similarity-based unsupervised spelling correction
